# Fibroblast Growth Factor Receptor-2 Expression in Thyroid Tumor Progression: Potential Diagnostic Application

**DOI:** 10.1371/journal.pone.0072224

**Published:** 2013-08-19

**Authors:** Adriano Redler, Giorgio Di Rocco, Domenico Giannotti, Francesca Frezzotti, Maria Giulia Bernieri, Simona Ceccarelli, Sirio D’Amici, Enrica Vescarelli, Anna Paola Mitterhofer, Antonio Angeloni, Cinzia Marchese

**Affiliations:** 1 Department of Surgical Sciences, Sapienza University of Rome, Rome, Italy; 2 Department of Radiological Sciences, Oncology and Anatomical Pathology, Sapienza University of Rome, Rome, Italy; 3 Department of Experimental Medicine, Sapienza University of Rome, Rome, Italy; 4 Department of Clinical Medicine, Sapienza University of Rome, Rome, Italy; 5 Department of Molecular Medicine, Sapienza University of Rome, Rome, Italy; Consiglio Nazionale delle Ricerche (CNR), Italy

## Abstract

Fibroblast growth factor receptor-2 (FGFR-2) plays an important role in tumorigenesis. In thyroid cancer it has been observed a FGFR-2 down-modulation, but the role of this receptor has not been yet clarified. Therefore, we decided to examine the expression of both FGFR-2 isoform, FGFR-2-IIIb and FGFR-2-IIIc, in different histological thyroid variants such as hyperplasia, follicular adenoma and papillary carcinoma. Immunohistochemistry and quantitative Real-Time PCR analyses were performed on samples of hyperplasia, follicular adenoma and papillary carcinoma, compared with normal thyroid tissue. Thyroid hyperplasia did not show statistically significant reduction in FGFR-2 protein and mRNA levels. Interestingly, in both follicular adenoma and papillary carcinoma samples we observed a strongly reduced expression of both FGFR-2 isoforms. We speculate that FGFR-2 down-modulation might be an early event in thyroid carcinogenesis. Furthermore, we suggest the potential use of FGFR-2 as an early marker for thyroid cancer diagnosis.

## Introduction

Thyroid cancer is the most common endocrine malignancy, with increasing incidence [1]. The most frequent thyroid tumor types are derived from follicular epithelial cells, which can be well differentiated in papillary thyroid carcinoma and follicular thyroid carcinoma, or poorly differentiated/undifferentiated in thyroid lethal anaplastic carcinoma [2]. These stages of differentiation have been correlated with a pattern of cumulative intragenic defects related with tumor differentiation, aggressiveness and metastatic potential [3]. Alterations in growth factors, such as MET, PDGF and VEGF, and their receptors, have been shown to be involved in thyroid tumor progression [3]. Also fibroblast growth factors (FGFs) and their receptors (FGFRs) have been implicated in the onset of thyroid carcinoma. These receptors have been shown to play a pivotal role in regulating cell proliferation, migration and differentiation during vertebrate development, as well as in response to injury and tissue repair [4]. The FGFRs family consists of four highly related genes, FGFR-1 to -4, encoding membrane receptors formed by two or three immunoglobulin-like extracellular domains, an intracellular tyrosine kinase domain, and a carboxyl-terminus domain [5]. FGFRs have been shown to be deregulated in malignancies of the brain [6], breast [7], prostate [8], skin [9], salivary gland [10], and thyroid gland [11]. FGFs and FGFRs are expressed in thyroid tissue [12], but an increased expression of FGFR-1 and FGFR-3 has been observed in benign and malignant thyroid tumors [13] and it has been implicated in the overgrowth of thyroid carcinoma cell lines [14]. FGFR-4 resulted to be strongly expressed in the more aggressive primary thyroid tumor [15] and its inhibition was able to induce apoptosis and cell cycle arrest in medullary thyroid cancer cells [16].

This work focuses on FGFR-2 gene, which is normally subjected to an alternative splicing that generates two cell type-specific isoforms, FGFR-2-IIIb/KGFR and FGFR-2-IIIc. FGFR-2-IIIb is expressed exclusively on epithelial cells of different organs while FGFR-2-IIIc is detected in mesenchymal cells. A shift between splicing isoforms may be involved in tumor progression. In rat prostate and bladder cancer models it has been indicated a switch from FGFR-2-IIIb to FGFR-2-IIIc isoform [17], [18], which might contribute to alter the balance between epithelial and mesenchymal cells and to promote epithelial-mesenchymal transition. However, FGFR-2-IIIb isoform re-expression in prostate and bladder cancer cell lines resulted in growth suppression *in vitro* and in decreased tumor formation *in vivo* [19]–[21]. FGFR-2 is known to be involved in the development of thyroid follicular gland. In particular, mice deficient for the FGFR-2-IIIb isoform show dysgenesis of thyroid, salivary and pituitary gland [22]. FGFR-2 is the only FGFR that is consistently expressed in normal thyroid tissue, whereas it appears to be reduced in thyroid cancers [15].

The aim of this study was the evaluation of a possible correlation between FGFR-2 expression and the pathological progression from normal thyroid tissue to thyroid carcinoma. Furthermore, we assessed the diagnostic value of FGFR-2 expression in benign and malignant thyroid lesions. To this aim, we investigated the expression of both FGFR-2 isoforms in thyroid hyperplastic tissues, thyroid follicular adenoma and papillary carcinoma by immunohistochemical procedures and Real-Time PCR analyses.

## Materials and Methods

### Ethics Statement

All experiments with human samples were conducted according to the principles expressed in the Declaration of Helsinki and approved by the Ethics Committee of the Azienda Policlinico Umberto I of Rome (official name of the committee). Following the Institutional Guidelines, written informed consent was obtained from all patients prior to their inclusion in the study.

### Tissue Collection

Thyroid samples were obtained from the Department of Surgical Sciences of the Sapienza University of Rome. We analyzed 8 male and 22 female patients, ranging in age from 35 to 71 years (mean age 53.6±7.2). The specimens included 6 nodular thyroid hyperplasia, 10 follicular thyroid adenomas and 14 papillary thyroid carcinomas. The normal control tissue was obtained for each patient from a portion of thyroid parenchyma without macroscopic alterations, contralateral to the lobe subjected to preoperative fine needle aspiration biopsy. Histopathological diagnosis of the tumors was made according to the World Health Organization criteria.

### Immunohistochemistry (IHC)

Small pieces of thyroid tissue were immediately fixed in 10% phosphate-buffered formalin solution, dehydrated through a graded ethanol series, cleared in xylene, and embedded in paraffin. Sections were cut on a sliding microtome, dewaxed with xylene and processed for immunostaining using LSAB+ System HRP (DakoCytomation, Inc., Carpinteria, CA, USA), followed by the addition of 3 - 3′ diaminobenzidine (DAB) as a chromogen. Endogenous peroxidase activity was quenched for 5 minutes in 3% hydrogen peroxidase, and the slides were rinsed in wash solution (TBST, 0.05 mol/L Tris Buffered Saline with Tween20). The antigen retrieval was performed with citrate buffer pH 6 for two times for 5 minutes in microwave. Then, the slides were placed into warm tap water, washed three times in phosphate-buffered saline (PBS; pH 7.4) for 5 minutes each and then incubated with blocking serum for 8 minutes. Immunostaining was performed using a home-made anti-FGFR-2-IIIb antibody at 1∶300 dilution for 1 hour at 25°C. The anti-FGFR-2-IIIb antibody used was a mouse monoclonal antibody raised against a peptide corresponding to amino acids 314–361 of the human FGFR-2-IIIb protein. Sections were counterstained with haematoxylin and photographed using a cooled CCD color digital camera SPOT-2 (Diagnostic Instruments Inc., Sterling Heights, MI, USA) and Axiovision software (Carl Zeiss, Oberkochen, Germany). Staining intensity was measured with the aid of NIH ImageJ v1.56 (National Institutes of Health, Bethesda, MD), as previously described [23].

Samples were scored based on the percentage of positive FGFR-2-IIIb area. Staining intensity was calculated for each category. Briefly, six patients for each group were processed and three images were taken from each patient. Mean values and standard deviations were obtained from five measurements of each image. The positive reaction was classified into three grades (0, 1, 2). Staining intensity was scored as grade 0 (negative) if 0–20% of tissue area showed positive reaction; grade 1 (moderate intensity) if 21–40% of tissue area showed positive reaction; grade 2 (strong positive) if >40% of tissue area showed positive reaction (Table 1).

**Table 1:** Classification of immunohistochemical grade and stain intensity according to the percentage of positive stained area.

Grade	Positive Area	Intensity
0	0–20%	Negative
1	21–40%	Moderate positive
2	>40%	Strong positive

### Quantitative Real-Time PCR

Thyroid samples were processed for total RNA extraction with the use of TRIzol reagent (Invitrogen, Karlsruhe, Germany). cDNA was generated with oligo (dT) from 1 µg of RNA using the SuperScript III Reverse Transcriptase Kit (Invitrogen). For FGFR-2-IIIb and FGFR-2-IIIc mRNA detection, specific custom TaqMan® Primer/Probe assays were developed (Table 2) and used at a concentration of 1× per well. A commercially available specific probe for FGFR-2 (Applied Biosystems by Life Technologies, Carlsbad, CA, USA) was used as a control. A total of 2 µl/well of template was added to the sample wells along with Taqman Universal PCR master mix at a concentration of 1× and water to a volume of 25 µl/well. Assays were performed in triplicate on an ABI 7500 Real-Time instrument (Applied Biosystems) using the following conditions: 50°C for 2 minutes, 95°C for 10 minutes, and then 95°C for 15 seconds and 60°C for 1 minute, repeated 40 times. Relative quantification was performed using GAPDH mRNA as an endogenous control: for each examined sample, FGFR-2, FGFR-2-IIIb or FGFR-2-IIIc mRNA expression data were normalized to the GAPDH expression.

**Table 2:** Custom TaqMan Assay gene-specific primers and reporter probes.

Gene	Forward Primer Sequence	Reverse Primer Sequence	Reporter Sequence
FGFR-2-IIIb	GGCTCTGTTCAATGTGACCGA	GTTGGCCTGCCCTATATAATTGGA	TTCCCCAGCATCCGCC
FGFR-2-IIIc	CACGGACAAAGAGATTGAGGTTCT	CCGCCAAGCACGTATATTCC	CCAGCGTCCTCAAAAG

### Construction of the FGFR-2-IIIb and FGFR-2-IIIc Standard Curve

To determine the absolute copy number of the target transcripts, a cloned plasmid DNA for FGFR-2-IIIb and FGFR-2-IIIc was used to generate a standard curve. The plasmid DNA was purified using Qiagen maxi prep kit (Qiagen, Chatsworth, CA, USA), according to the manufacture’s directions. FGFR-2-IIIb and FGFR-2-IIIc plasmids contain 76 and 77 bp of cDNA inserts, respectively, in a pJET1.2/blunt vector (2974 bp) (Fermentas, Vilnius, Lithuania). The copy numbers of plasmid DNA template were calculated according to the molecular weight of the plasmid and then converted into the copy numbers based upon the Avogadro’s number. Each cloned plasmid DNA was serially (every fivefold) diluted at a range of 5.0×10^6^–3.2×10^2^ copy numbers. Each sample was run in triplicates.

### Absolute Quantitation of FGFR-2-IIIb and FGFR-2-IIIc by Real-Time PCR

Amplification conditions were 50°C for 2 minutes, 95°C for 10 minutes, and then 95°C for 15 seconds and 60°C for 1 minute, repeated 40 times. All reactions were performed on an ABI 7500 Real-Time instrument (Applied Biosystems). A standard curve was constructed by plotting the threshold cycle (CT) versus the known copy numbers of the template in the standard. According to the standard curve, the copy numbers for all unknown samples were obtained automatically.

The absolute copy number of FGFR-2-IIIb and FGFR-2-IIIc mRNA in each sample was calculated based on its C_T_ value with its plasmid DNA standard curve, and then normalized to GAPDH to minimize variability in the results due to differences in the reaction efficiency and RNA integrity among test samples.

### Statistical Analysis

Each set of experiments was repeated at least in triplicate, and standard deviation values were calculated. Student’s two-tailed *t*-test was used for statistical analysis, and *P*-values less than 0.05 were considered statistically significant.

## Results

### FGFR-2-IIIb Expression in Thyroid Tumor Progression

**Figure 1:**
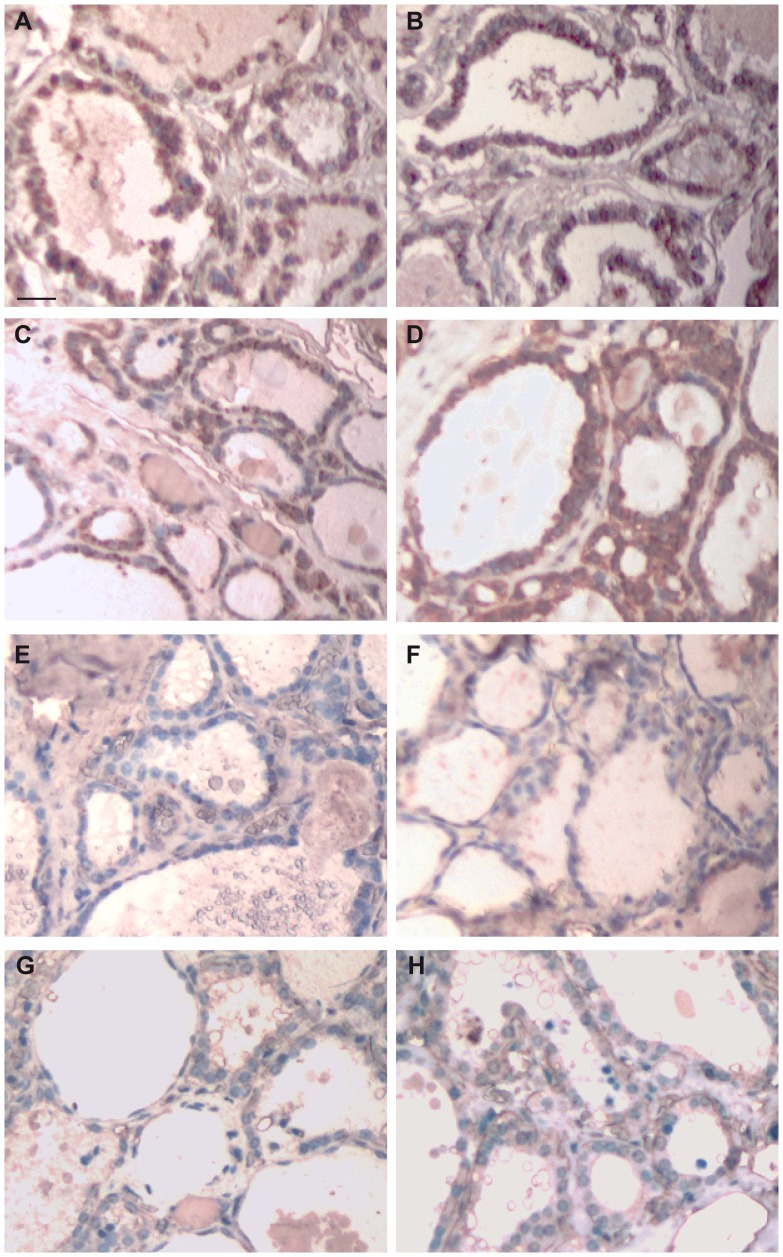
Immunohistochemical detection of FGFR-2-IIIb in thyroid tissue. FGFR-2-IIIb immunohistochemical detection was performed with a home-made antibody on sections of normal thyroid tissue (**A**, **B**), hyperplastic thyroid tissue (**C**, **D**), follicular adenoma (**E**, **F**) and papillary carcinoma (**G**, **H**). Representative tissue sections for each group are shown (original magnification 10×, scale bar 20 µm).

**Figure 2:**
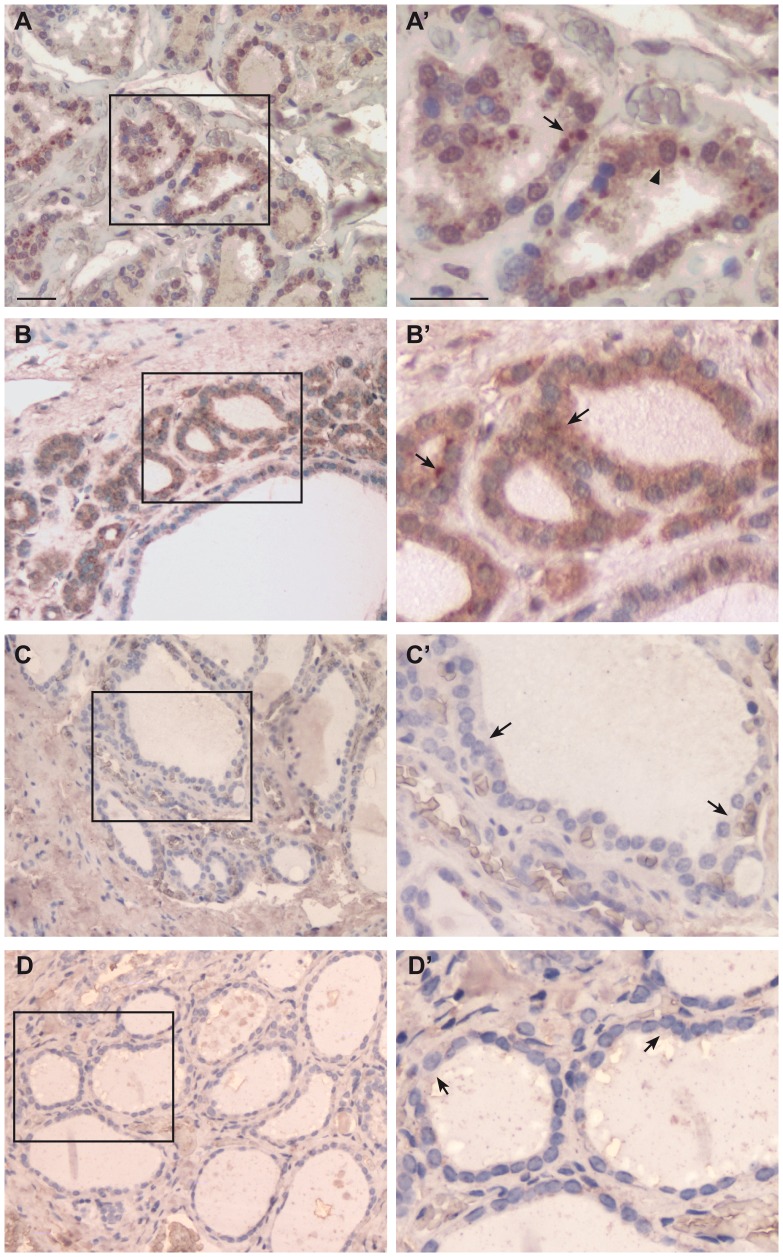
Immunohistochemical detection of FGFR-2-IIIb in thyroid tissue. Dewaxed sections of normal thyroid tissue (A, A’), hyperplastic thyroid tissue (B, B’), follicular adenoma (C, C’) and papillary carcinoma (D, D’) were subjected to immunohistochemistry using a home-made antibody directed against FGFR-2-IIIb. (**A–D**), original magnification 10×, scale bar 20 µm. A significant area in each panel is indicated by a square and an enlargement of this area is shown in the respective (**A’–D’**) panels (original magnification 20×, scale bar 20 µm). (**A–A’**) Normal thyroid sections showing positive staining pattern for FGFR-2-IIIb in both cytoplasm (arrow) and cell membrane compartment (arrowhead) of follicular cells. (**B–B’**) Hyperplastic thyroid sections showing positive staining pattern for FGFR-2-IIIb in follicular cells cytoplasm (arrows). (**C–C’**)**,** (**D–D’**) Follicular adenoma and papillary carcinoma samples showing no FGFR-2-IIIb signal in follicular cells (arrows).

FGFR-2-IIIb expression was investigated by IHC in three different pathological thyroid samples and compared to that of normal thyroid tissue, with the aid of a home-made FGFR-2-IIIb-specific monoclonal antibody. The functionality of our antibody and its specificity for the FGFR-2-IIIb isoform was assessed by immunofluorescence and Western blot analysis on both epithelial and mesenchymal cells (human keratinocytes and human fibroblasts, respectively). As shown in figure S1, our antibody recognized specifically the IIIb isoform on epithelial cells, while no signal was observed in mesenchymal cells, thus demonstrating that the antibody does not react with the IIIc isoform. Normal thyroid tissue showed positive staining for FGFR-2-IIIb (Fig. 1, panels A, B), in accordance with previous literature describing FGFR-2 expression in this organ. In particular, in normal thyroid gland FGFR-2-IIIb staining was localized focally to follicular epithelial cells (Fig. 2, panels A, A’), with intense expression on cell membranes (arrowhead in panel A’) and cytoplasm (arrow in panel A’). Quantification of IHC signal, measured by means of ImageJ software, revealed a percentage of positive FGFR-2-IIIb area of 41.7±2.7. According to our classification (Table 1), normal tissue resulted to be strongly positive for FGFR-2-IIIb (grade 2).

We observed a slight decrease of FGFR-2-IIIb staining intensity in hyperplastic thyroid tissue (Fig. 1, panels C, D), which is still well represented in the cytoplasm of follicular epithelial cells (Fig. 2, panels B, B’, arrows in panel B’). In these samples, the percentage of positively stained area was 35.3±7.4, corresponding to a moderately positive signal intensity (grade 1) (*P* = 0.09 *vs* normal tissue). In contrast, in follicular adenoma (Fig. 1, panels E, F), we observed a significant reduction in FGFR-2-IIIb staining intensity, with no evident signal both in cell membrane and cytoplasm (Fig. 2, panels C, C’, arrows in panel C’). Analysis software confirmed a drastic reduction in FGFR-2-IIIb staining intensity in adenoma (15.7±4.8), corresponding to grade 0 (*P*<0.01 *vs* normal tissue). Also in papillary carcinoma (Fig. 1, panels G, H), we could observe no FGFR-2-IIIb staining in cytoplasm or membrane (Fig. 2, panels D, D’, arrows in panel D’). Indeed, IHC quantitative evaluation indicated that the percentage of positive FGFR-2-IIIb staining area in carcinoma samples was 12.8±2.5, thus corresponding to negative signal (grade 0) (*P*<0.01 *vs* normal tissue).

As concerning FGFR-2-IIIc expression, we could not evaluate it by IHC given the lack of specific antibodies. Nevertheless, we performed a set of IHC experiments using the commercially available Bek antibody, which recognizes both FGFR-2 isoforms, but it was not able to detect significant variations due to poor sensitivity and high background (data not shown).

### FGFR-2 mRNA Expression Levels in Thyroid Tumor Progression

**Figure 3:**
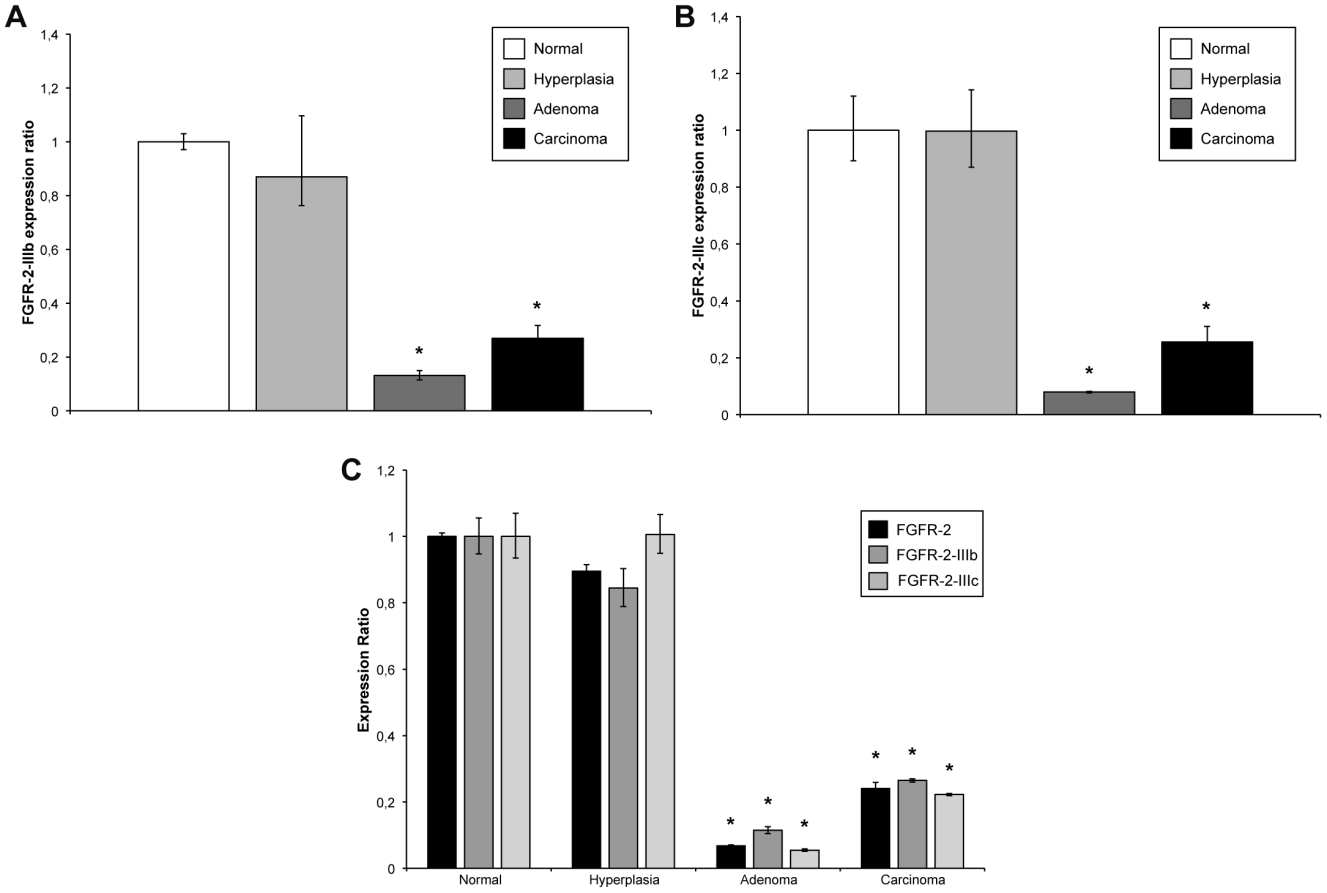
FGFR-2, FGFR-2-IIIb and FGFR-2-IIIc mRNA expression in thyroid cancer progression. (**A**) Quantitative Real-Time PCR analysis of FGFR-2-IIIb mRNA expression in normal thyroid tissue, hyperplastic thyroid tissue, follicular adenoma and papillary carcinoma. Relative FGFR-2-IIIb mRNA levels are shown as fold value of the level of FGFR-2-IIIb in normal tissue. mRNA levels were normalized to GAPDH mRNA expression. Error bars represent standard deviations. *P<0.01. (**B**) Quantitative Real-Time PCR analysis of FGFR-2-IIIc mRNA expression in normal thyroid tissue, hyperplastic thyroid tissue, follicular adenoma and papillary carcinoma. Relative FGFR-2-IIIc mRNA levels are shown as fold value of the level of FGFR-2-IIIc in normal tissue. mRNA levels were normalized to GAPDH mRNA expression. Error bars represent standard deviations. *P<0.01. (**C**) Quantitative Real-Time PCR analysis of FGFR-2, FGFR-2-IIIb and FGFR-2-IIIc mRNA expression in normal thyroid tissue, hyperplastic thyroid tissue, follicular adenoma and papillary carcinoma. Relative FGFR-2, FGFR-2-IIIb and FGFR-2-IIIc mRNA levels are shown as fold value of the level of each gene in normal tissue. mRNA levels were normalized to GAPDH mRNA expression. Error bars represent standard deviations. *P<0.01.

**Figure 4:**
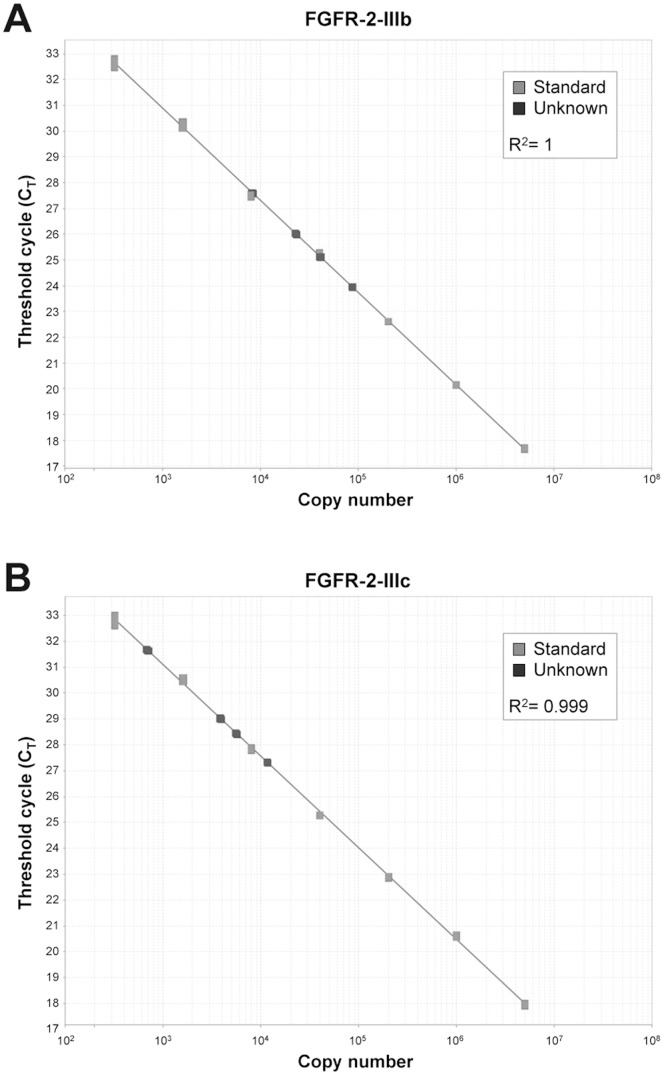
Absolute quantitation of FGFR-2-IIIb and FGFR-2-IIIc mRNA in thyroid cancer progression. Real-time PCR of FGFR-2-IIIb (**A**) and FGFR-2-IIIc (**B**) standard curves using a cloned plasmid DNA as a template. Serial dilutions of the plasmid DNA were used as the templates. C_T_ (threshold cycle) represents the PCR cycle at which the reporter fluorescence above the baseline signal can be detected. The standard curves plot the input DNA templates copy numbers against the C_T_ values (grey squares). All of our testing samples were within the standard range (black squares). The correlation coefficients R^2^ indicate the closeness of fit between the regression line and the individual C_T_ data points of the standard reactions.

The modulation of FGFR-2 expression in thyroid tissue was confirmed at mRNA level by quantitative Real-Time PCR. As concerning FGFR-2-IIIb, in hyperplastic thyroid samples we did not observe a significant decrease with respect to normal tissue (0.87 fold, *P* = 0.14), while both adenoma and carcinoma samples showed a consistent reduction of FGFR-2-IIIb expression (0.13 and 0.27 fold, respectively, *P*<0.01) (Fig. 3A). Similar results were obtained for FGFR-2-IIIc, with no variation in mRNA expression for hyperplastic tissue (0.99 fold, *P* = 0.94) and strong decrease in adenoma (0.08 fold, *P*<0.01) and carcinoma (0.26 fold, *P*<0.01) samples (Fig. 3B). We also performed Real-Time PCR analysis to evaluate total FGFR-2 mRNA levels in thyroid tissue (Fig. 3C). Such analysis provided the confirmation of FGFR-2 expression trend in thyroid samples (0.89 fold in hyperplastic tissue, *P = *0.07; 0.07 fold in adenoma samples, *P*<0.01; 0.24 fold in carcinoma samples, *P*<0.01). Then, to allow the evaluation of the relative contribution of FGFR-2-III-b and FGFR-2-IIIc in thyroid samples, we performed an absolute quantification by Real-Time PCR. In detail, using a series of diluted plasmid DNA as templates, a standard curve for FGFR-2-IIIb (Fig. 4A) and FGFR-2-IIIc (Fig. 4B) was obtained. Since the C_T_ value decreases linearly with the increasing amount of plasmid DNA copy number and all of our tested samples (healthy tissue, hyperplasia, adenoma and carcinoma) were located within this linear amplification range (Fig. 4A, B), the copy number of FGFR-2-IIIb and FGFR-2-IIIc transcripts in our test samples could be measured by using the standard curve.

Because the measurement of RNA concentration is often inaccurate, the difference among RNA samples was corrected using GAPDH as internal control housekeeping gene. The normalized quantitative data of FGFR-2-IIIb and FGFR-2-IIIc mRNA expression in thyroid samples, reported in Table 3, indicate that the FGFR-2-IIIb isoform is much more expressed than the FGFR-2-IIIc. In particular, the relative contribution of the two isoforms to the total FGFR-2 in thyroid tissue is between 86 and 92% for FGFR-2-IIIb and between 8 and 14% for FGFR-2-IIIc (see Table 3).

**Table 3:** Absolute quantitation of FGFR-2-IIIb and FGFR-2-IIIc mRNA in thyroid samples by Real-Time PCR.

Thyroid sample	FGFR-2-IIIb copies	Percentage of totalFGFR-2	FGFR-2-IIIc copies	Percentage of totalFGFR-2
Normal	8.53 × 10^4^	88%	1.17 × 10^4^	12%
Hyperplasia	6.87 × 10^4^	86%	1.16 × 10^4^	14%
Adenoma	1.01 × 10^4^	92%	8.45 × 10^2^	8%
Carcinoma	2.16 × 10^4^	88%	2.97 × 10^3^	12%

## Discussion

Thyroid tumor is the most common malignant cancer of endocrinology, with an increasing incidence in the world. In the lack of specific markers of malignancy, it is not always possible to differentiate between benign and malignant thyroid diseases. In this work, we investigated the expression of FGFR-2 in different thyroid normal and pathological tissue samples, in order to evaluate its potential use as a diagnostic marker. The choice of FGFR-2 is justified by its role in the formation of thyroid follicular gland [22] and its suggested involvement in thyroid tumor progression. In particular, it is known that normal human thyroid tissue expresses FGFR-2, while it is not detectable in thyroid tumor cell lines [24]. Down-regulation of FGFR-2 has been also reported in other human neoplasms, including astrocytomas, bladder and prostate carcinomas, pituitary adenomas [20], [25], [26], and FGFR-2 loss-of-function mutations have been identified in melanoma [27]. Moreover, previous studies suggested a protective role of FGFR-2 against cancer progression in transformed thyroid carcinoma cells [15], [28]. FGFR-2 down-modulation in thyroid cancer has been justified as a result of DNA promoter methylation of FGFR-2 gene. The subsequent FGFR-2 re-expression seemed to reduce thyroid cancer progression by enhancing apoptosis in tumoral cells [29]. Nevertheless, the complex role of FGFR-2 gene in carcinogenesis, and in particular of the selective expression of FGFR-2-IIIb or FGFR-2-IIIc isoform, is still not well understood.

In light of these considerations, we decided to assess FGFR-2 expression evaluating their relevance in thyroid tumors progression and diagnosis. In this study, we first analyzed FGFR-2-IIIb expression in thyroid samples by means of IHC. We were able to confirm that normal thyroid tissue is strongly positive for FGFR-2-IIIb staining, while both benign and malignant thyroid alterations showed negative staining. Interestingly, we observed only a slight reduction of FGFR-2-IIIb expression in thyroid hyperplasia, which retained a moderate positivity to this receptor. To further confirm these data and to better address FGFR-2-IIIc expression, which could not be evaluated by IHC due to the lack of specific antibodies, we decided to switch to molecular analysis because it allowed us to selectively analyze the expression of both FGFR-2 isoforms. Moreover, the molecular approach with Real-Time PCR allowed us to obtain a quantitative evaluation, which is more reliable than that obtained through ImageJ.

We confirmed through Real-Time PCR analysis a relevant down-modulation of both FGFR-2 isoforms in follicular adenoma and papillary carcinoma, with respect to normal thyroid tissue. Such decrease suggests that FGFR-2 down-modulation may be a critical step in thyroid cancer progression, and it can be assumed that FGFR-2 alteration may be an early event in the development of thyroid neoplasia.

Real-Time PCR analysis clearly confirmed the striking difference between hyperplasia and neoplastic lesions in terms of FGFR-2 expression. In fact, both FGFR-2-IIIb and FGFR-2-IIIc transcript levels, while strongly down-modulated in adenoma and carcinoma, were virtually unchanged in hyperplasia with respect to normal tissue.

Moreover, we were able to perform an absolute quantification of the two isoforms by Real-Time PCR, thus demonstrating for the first time their relative contribution in thyroid tissue.

Diagnosis of thyroid cancer typically involves a procedure known as “Fine Needle Aspiration”, in which thyroid cells obtained from one or more nodules are observed and morphologically evaluated. The reliability of this cytological diagnosis is still limited by the reduced number of cells available for the analysis and by the variability due to the operator experience. Therefore, the introduction of immunocytological markers could be useful for discriminating between hyperplastic proliferation and neoplastic transformation.

The present study is aimed to update information about FGFR-2 expression in different histological thyroid types and its potential correlation with thyroid tumor progression. The differential expression profile of FGFR-2, identified by both IHC and Real-Time PCR, between normal/hyperplastic and benign/malignant conditions, might suggest the adoption of this receptor as a diagnostic marker for thyroid tissue. Future studies will be aimed to consolidate our observations by increasing the number of patients, and to assess the feasibility of FGFR-2 staining also on cytological samples derived from fine needle aspiration, in order to introduce the use of this marker in the routine procedures for thyroid diseases diagnosis.

## Supporting Information

Figure S1 **Functionality and specificity of home-made FGFR-2-IIIb antibody.** A) Immunofluorescence analysis on HK cells (panel a), HF cells (panel b) and HF cells transiently transfected with KGFR (panels c, d) with home-made FGFR-2-IIIb-specific monoclonal antibody followed by the appropriate FITC-conjugated secondary antibody (green). Scale bar 20 µm. B) Western blotting analysis of FGFR-2-IIIb protein levels in HK and HF cells. FGFR-2-IIIb protein expression was evaluated by blotting with our FGFR-2-IIIb-specific monoclonal antibody. Anti-Tubulin antibody was used as loading control. The images are representative of at least three independent experiments.
(TIF)

Text S1 **Supporting Materials and Methods.**
(DOC)
