# Supplementary figures and images for: Fibroblast Growth Factor Receptor-2 Expression in Thyroid Tumor Progression: Potential Diagnostic Application
Source: PLoS One. 2013 Aug 19;8(8):e72224. doi: 10.1371/journal.pone.0072224 (PMC3747152; doi:10.1371/journal.pone.0072224)

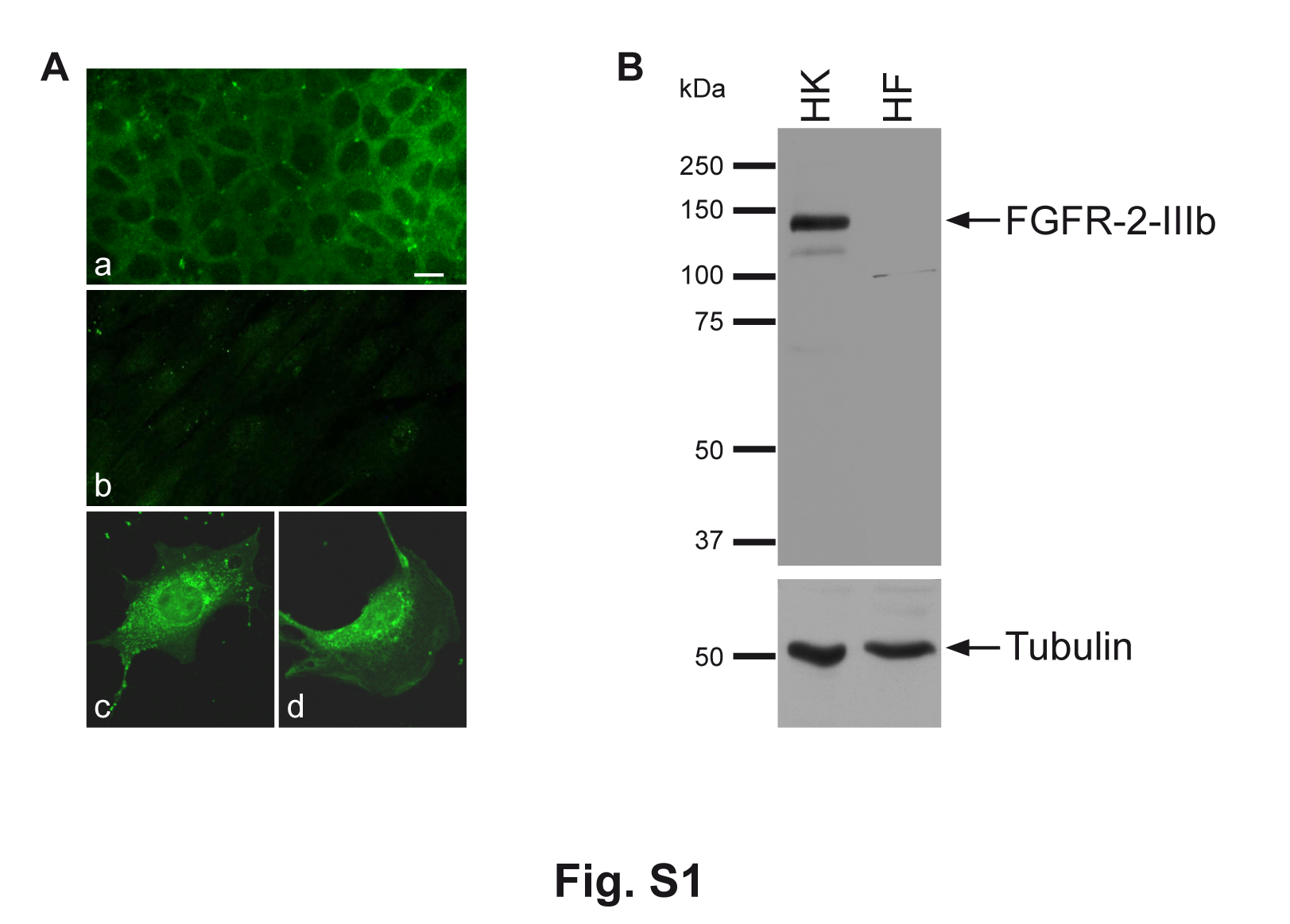

Supplement: Figure S1 — Functionality and specificity of home-made FGFR-2-IIIb antibody. A) Immunofluorescence analysis on HK cells (panel a), HF cells (panel b) and HF cells transiently transfected with KGFR (panels c, d) with home-made FGFR-2-IIIb-specific monoclonal antibody followed by the appropriate FITC-conjugated secondary antibody (green). Scale bar 20 µm. B) Western blotting analysis of FGFR-2-IIIb protein levels in HK and HF cells. FGFR-2-IIIb protein expression was evaluated by blotting with our FGFR-2-IIIb-specific monoclonal antibody. Anti-Tubulin antibody was used as loading control. The images are representative of at least three independent experiments. (TIF) [file pone.0072224.s001.tif]
