# Supplementary material for: Fibroblast Growth Factor Receptor-2 Expression in Thyroid Tumor Progression: Potential Diagnostic Application
Source: PLoS One. 2013 Aug 19;8(8):e72224. doi: 10.1371/journal.pone.0072224 (PMC3747152; doi:10.1371/journal.pone.0072224)
Supplement: Text S1 — Supporting Materials and Methods. (DOC) [file pone.0072224.s002.doc]

**SUPPORTING MATERIALS AND METHODS**

**Reagents and cell cultures**

Primary cultures of human keratinocytes (HK) and human fibroblasts (HF) were established from 1 cm^2^ full-thickness skin biopsy from a healthy donor and maintained in chemical defined medium KGM (Lonza Milano S.r.l., Milan, Italy), and in DMEM containing 10% FBS, respectively, with medium change twice a week.

HF cells, grown in coverslips onto 24 plates, were transiently transfected with the pCEV27 vector containing human KGFR cDNA (kindly provided by Dr Stuart A. Aaronson, New York, New York).

**Immunofluorescence microscopy**

HK, HF and HF-KGFR, grown on coverslips, were fixed in 4% paraformaldehyde in phosphate-buffered saline (PBS) for 30 min at 25°C, followed by treatment with 0.1 M glycine in PBS for 20 min at 25°C and with 0.1% Triton X-100 in PBS for additional 5 min at 25°C to allow permeabilization. Then, cells were incubated with home-made FGFR-2-IIIb-specific monoclonal antibody (1:100 in PBS). Primary antibody was visualized using goat anti-mouse FITC-conjugated IgG (1:50 in PBS; Jackson ImmunoResearch Laboratories, West Grove, PA, USA).

**Western blot analysis**

HK and HF cells were lysed in RIPA buffer. Total proteins (50 μg) were resolved under reducing conditions by 8% SDS–PAGE and transferred to Immobilon-FL membranes (Millipore). Membranes were incubated overnight at 4 °C with home-made FGFR-2-IIIb-specific monoclonal antibody (1:1000), followed by goat anti-mouse horseradish peroxidase (HRP)-conjugated secondary antibody (Sigma-Aldrich). To estimate the protein equal loading, the membranes were rehydrated, stripped and reprobed with anti-tubulin

antibodies diluted 1:2000. Bound antibody was detected by enhanced chemiluminescence detection reagents (Pierce Biotechnology Inc, Rockford, IL, USA), according to manufacturer’s instructions.
